# Trends in waterpipe and e-cigarette use among young people in the European Union, 2012–2023: a repeated cross-sectional study

**DOI:** 10.1016/j.lanepe.2026.101815

**Published:** 2026-08-13

**Authors:** Mui Siew Tan, Filippos T. Filippidis, Charlotte Xin Li, Anthony A. Laverty, Esteve Fernández, Cristina Martínez, Armando Peruga, Constantine I. Vardavas, Ariadna Feliu

**Affiliations:** a Department of Primary Care and Public Health, School of Public Health, Imperial College London, London, United Kingdom; b European Network for Smoking and Tobacco Prevention, Brussels, Belgium; c Tobacco Control Unit, Cancer Control and Prevention Program, WHO Collaborating Center on Tobacco Control, Institut Català d’Oncologia, L’Hospitalet de Llobregat, Spain; d Cancer Control and Prevention Group, Institut d’Investigació Biomèdica de Bellvitge-IDIBELL, L’Hospitalet de Llobregat, Spain; e CIBER en Enfermedades Respiratorias (CIBERES), Madrid, Spain; f School of Medicine and Health Sciences, Campus de Bellvitge, Universitat de Barcelona, L'Hospitalet del Llobregat, Spain; g Secretariat of Public Health, Department of Health, Generalitat de Catalunya, Barcelona, Spain; h Department of Public Health, Mental Health, and Maternal and Child Health Nursing, School of Nursing –Bellvitge Campus, Universitat de Barcelona, L'Hospitalet del Llobregat, Spain; i Philip R. Lee Institute for Health Policy Studies, University of California San Francisco, San Francisco, CA 94158, USA; j Centro de Epidemiología y Políticas de Salud, Facultad de Medicina - Clínica Alemana, Universidad del Desarrollo, Santiago, Chile; k Department of Preventive Medicine, Epidemiology and Medical Statistics, School of Medicine, National and Kapodistrian University of Athens, Athens, Greece

**Keywords:** Waterpipe, E-cigarette, Prevalence, Youth, Trends, European Union

## Abstract

**Background:**

Alternative tobacco and nicotine-containing products can challenge progress in tobacco control. Waterpipes and e-cigarettes gained popularity among youth at different times, driven by flavours, social influence, and perceived reduced harm. This study examined trends in waterpipe and e-cigarette use among youth in the European Union (EU) from 2012 to 2023.

**Methods:**

We analysed data from five waves (2012–2023) of the Special Eurobarometer surveys in 26 EU Member States (N = 20,466; ages 15–30). Multilevel Poisson models assessed trends by sociodemographic subgroups, adjusting for country-level factors. Adjusted prevalence ratios (aPRs) with 95% confidence intervals (CIs) are reported.

**Findings:**

From 2012 to 2023, ever waterpipe use fell from 31.2% (95% CI: 29.1–33.3%) to 14.9% (13.2–16.8%), and current use (since 2017) from 2.5% (1.8–3.5%) to 1.8% (1.2–2.7%). Ever e-cigarette use rose from 12.2% (10.8–13.8%) to 25.8% (23.7–28.2%), and current use from 1.6% (1.1–2.3%) to 5.3% (4.0–6.8%), with cross-country variation. Compared with 2017, ever waterpipe use was lower in 2023 (aPR: 0.52, 95%CI: 0.42–0.66). Ever e-cigarette use was lower in 2012 (0.47; 0.40–0.54) and 2014 (0.75; 0.67–0.84). Current waterpipe use increased in 2020 (1.96; 1.16–3.28) then declined, while current e-cigarette use rose in 2020 (1.64; 1.15–2.34) and 2023 (4.27; 2.73–6.67). By 2023, ever waterpipe use was lower across all subgroups despite earlier heterogeneity, whereas ever e-cigarette use was higher among women (1.41; 1.18–1.69), urban residents (1.27; 1.11–1.46), and those without financial difficulties (1.38; 1.20–1.60). Current e-cigarette use was also higher across all groups.

**Interpretation:**

Declining waterpipe use and rising e-cigarette use among young Europeans show divergent patterns and differences across sociodemographic groups, particularly among women and socioeconomically disadvantaged groups. Continued surveillance and targeted interventions are needed to address emerging vulnerabilities in high-risk populations.

**Funding:**

European Union’s Horizon 2020.

**Research in context**

**Evidence before this study**

Waterpipes and e-cigarettes have become popular among young people in the European Union (EU) and may facilitate nicotine initiation, including among never-smokers, through features such as appealing flavours, high social acceptability, extensive marketing, and perceived reduced harm.

As most people who smoke initiate between ages 14 and 25, monitoring these products is essential. We searched PubMed for English-language articles published from inception to Aug 1, 2025, using the terms (“waterpipe” OR “shisha” OR “hookah” OR “narghile” OR “e-cigarettes” OR “vape” OR “electronic cigarette”) AND (“young people” OR “youth” OR “adolescent”). Although previous studies have described trends among EU adults, evidence on temporal trends and sociodemographic differences among young people is limited.

**Added value of this study**

This is the first study using regionally representative EU data to examine trends in waterpipe and e-cigarette use among EU youth over more than a decade and across sociodemographic groups. Ever waterpipe use declined, whereas both ever and current e-cigarette use increased steadily. Increases in e-cigarette use differed by gender and financial circumstance, suggesting widening disparities. Our findings provide comparable evidence across countries and survey waves to inform EU tobacco and nicotine control polices.

**Implications of all the available evidence**

Together with previous research, our findings suggest that, despite declining waterpipe use, e-cigarettes are driving increasing nicotine use among EU youth. Continued standardised surveillance and targeted gender- and equity-sensitive policies regulating product characteristics, pricing, marketing, and availability are needed to support the EU tobacco endgame.

## Introduction

The European Union (EU) has made substantial progress in tobacco control. Over the past two decades, smoking prevalence declined from 39.4% in 2002 to 24.0% in 2023.^1^^,^^2^ Nevertheless, tobacco use remains a major public health burden, causing over 700,000 premature deaths annually and accounting for about 27% of cancer cases in the region.^3^^,^^4^ The emergence and rapid evolution of alternative tobacco and nicotine-containing products complicate efforts to achieve further reductions in cigarette smoking prevalence. Although manufactured cigarettes retain market dominance, non-cigarette products, such as waterpipes and e-cigarettes, increasingly shape the tobacco and nicotine market.^5^

The EU and its Member States (MS) have ratified the World Health Organization Framework Convention on Tobacco Control (WHO FCTC) and implemented MPOWER measures to reduce tobacco use, including systematic monitoring of consumption.^6^^,^^7^ In 2021, the EU launched the Europe’s Beating Cancer Plan and set the goal of a ‘Tobacco-Free Generation’ in which <5% of the population uses tobacco.^4^ Young people are central to this goal, as most people who smoke initiate use between ages 14 and 25, and earlier initiation is associated with greater nicotine dependence and lower likelihood of cessation.^8^^,^^9^

Waterpipe and e-cigarettes share characteristics that make them particularly appealing to young people, including flavours, social acceptability, media influence, and perceived reduced harm.^10^ These features have contributed to their uptake among adolescents and young adults, including those who have never smoked. Although their historical trajectories differ, waterpipe use expanded globally in the 1990s, whereas e-cigarettes emerged and spread rapidly in the early 2010s,^11^ both products have become important sources of nicotine experimentation and recreational use among young people, and are therefore epidemiologically comparable in their potential behavioural roles in population-level patterns of experimentation, even if their determinants of uptake and risk profiles may differ.

Despite their shared appeal, waterpipes and e-cigarettes differ in composition and framing. Waterpipes are traditional tobacco products for recreational use, whereas e-cigarettes are more recent devices that typically do not contain tobacco and are sometimes considered within harm reduction or smoking cessation frameworks. However, both products are associated with adverse health outcomes, including cancer, respiratory, and cardiovascular diseases.^12^ Both expose users to nicotine and contribute to dependence and long-term addiction,^13^ with growing evidence linking their use to subsequent cigarette smoking initiation.^14^^,^^15^ In this context, our interest is not in equating their toxicological risks, but in examining how two distinct product types are used within population-level patterns of nicotine experimentation and recreational use among youth.

In the EU, both waterpipe and e-cigarette use increased among adults between 2012 and 2014, but diverged thereafter: waterpipe use declined while e-cigarette use continued to rise.^16^, ^17^, ^18^ This divergence may reflect shifts in product appeal, availability, and promotion, raising the possibility that e-cigarettes are increasingly associated with patterns of nicotine use among young people that co-occur with those observed for waterpipes. This raises the hypothesis of a potential “cohort substitution effect”, whereby e-cigarettes may be progressively replacing waterpipes as a preferred vehicle for nicotine experimentation among younger cohorts, even if such transitions are not frequently observed at the individual level. While previous EU-wide analyses reported that young people had higher odds of trying these products compared with older adults,^17^^,^^18^ it remains unclear whether trends among young people mirror those observed in the broader adult population, as existing studies vary methodologically and lack comparability over time.

To address this gap, this study examines trends in waterpipe and e-cigarette use among young people (15–30 years) in the EU between 2012 and 2023, focusing on how the prevalence of each product has evolved over time, and explores differences across sociodemographic groups. By analysing waterpipes and e-cigarettes in parallel, we aim to characterise their joint and divergent population-level patterns of use among young people and to assess whether changes in e-cigarette use co-occur temporally with changes in waterpipe use in this age group.

## Methods

### Study design

This study used repeated cross-sectional study from five waves of the Special Eurobarometer surveys on tobacco (waves 77.1 [2012], 82.4 [2014], 87.1 [2017], 93.2 [2020], and 99.3 [2023]), conducted by the European Commission and designed to be nationally representative of individuals aged ≥15 years in each EU MS.^19^ Sample sizes were approximately 1000 respondents per country, with smaller samples of around 500 respondents, in countries such as Cyprus, Luxembourg, and Malta. Twenty-six EU MS were included; Croatia and the United Kingdom were excluded to ensure consistency across waves, as they were not covered in the first and last survey years, respectively.

The Eurobarometer surveys use a standardised multistage random sampling method. Primary Sampling Units (PSUs) are selected with probability proportional to population size and stratified by region and degree of urbanisation, ensuring coverage of all Eurostat NUTS II regions as well as metropolitan, urban, and rural areas. Within each PSU, addresses are selected using a combination of random starting points and systematic sampling procedures. One individual per household is then selected using the nearest birthday rule and interviewed in the national language. Post-stratification weights provided by Eurostat were applied to align samples with national and EU population distributions by age, sex, and area of residence. Further methodological details are available elsewhere.^19^

The 2020 wave was conducted under COVID-19 pandemic conditions, which led to deviations in fieldwork procedures, including disruptions to standard face-to-face data collection and related adaptations in fieldwork timing and logistics, with several countries, such as Belgium, Denmark, Estonia, Finland, Ireland, Luxembourg, Netherlands, Spain, and Sweden, switching partially or entirely to online computer-assisted web interviewing.

We defined young people as individuals between 15 and 30 year of age. The analytic sample of this study with complete cases included a total of 20,466 respondents across 26 EU MS (Supplementary Fig. S1).

### Outcome measures

#### Ever use

Ever use of waterpipe and e-cigarettes was used as an indicator of lifetime experimentation or exposure and early adoption of these products. All participants were asked if they had ever tried waterpipe. In 2012 and 2014, responses included “Yes, you use or used it regularly”; “Yes, you use or used it occasionally”; “Yes, you tried it once or twice; “No”; and “Don’t Know”. Responses starting with “Yes” were considered as ever use of waterpipe. From 2017, responses were frequency-based, including “Every day”; “Every week”; “Every month”; “Less than monthly”; “You used to use it regularly, but you have stopped”; “You have tried it only once or twice”; “Never”; “Refusal”; and “Don’t know”. Individuals selecting any of the first six responses were classified as ever users of waterpipe.

All participants were asked if they had tried e-cigarettes. In 2012, the responses included “Yes, you use or used it regularly”; “Yes, you use or used it occasionally”; “Yes, you tried it once or twice”; “No”; and “Don’t know”. The first three responses were considered as ever use of e-cigarettes. From 2014 onwards, responses (with slight variations in wording) were “You currently use electronic cigarettes or similar electronic devices (e.g., e-shisha, e-pipe)”; “You used them in the past but no longer use them”; “You tried them in the past but no longer use them”; “You have never used them”; and “Don’t know”. Individuals selecting any of the first three responses were classified as ever users of e-cigarettes.

The survey questions, response options, and definitions used to classify waterpipe and e-cigarette ever use across Eurobarometer waves are summarised in Supplementary Table S1.

#### Current use

We defined current use of waterpipe or e-cigarettes as use at least monthly at the time of data collection, as an indicator of recent, ongoing and socially patterned behaviour rather than established or dependent use. Analyses were limited to waves 2017–2023 due to non-comparable responses in earlier waves.

Current waterpipe use was derived from the frequency-based responses used to assess ever use: individuals reporting use at least monthly were classified as current users. For e-cigarettes, respondents who reported that they “currently use” the product were asked about frequency of use, with response options including “Every day”; “Every week”; “Every month”; “Less than monthly”; “You have tried only once or twice”; “Never”; and “Refusal”. Those reporting e-cigarettes use at least monthly were classified as current users.

### Covariates

#### Individual-level variables

##### Sociodemographic characteristics

Data were collected on participants’ age, gender, area of residence and financial circumstance. We categorised age as 15–17, 18–24 and 25–30 years. Gender was recorded as man or woman in 2012–2017; from 2020 onwards, participants could also select “None of the above/Non-binary/Do not recognise yourself in the above categories”. To ensure comparability across waves, analyses were restricted to respondents identifying as man and woman (0.09% excluded). Area of residence was classified as rural if “rural area or village”, and urban if “town” or “city” was selected. Responses on difficulty paying bills in the last 12 months were used as a proxy for experiencing financial difficulty and grouped into two categories: almost never/never and most of the time/from time to time. Descriptive statistics for these variables are presented in Supplementary Tables S2 and S3.

##### Smoking status

Smoking status was derived from responses to the question: *“Regarding smoking cigarettes, cigars, cigarillos, or a pipe, which of the following applies to you?”*. Respondents reporting “You currently smoke” were classified as current smokers, those reporting “You used to smoke but you have stopped” as former smokers, and those reporting “You have never smoked” as never smokers. The question wording and response options were consistent across all survey waves.

#### Country-level variables

##### Actual Individual Consumption (AIC) per capita

AIC per capita measures the value of all goods and services consumed per person in a country, including household purchases and services. It was used to adjust for underlying differences in living standard and purchasing power across EU. Data for each country and survey year were obtained from Eurostat.^20^

##### Tobacco Control Scale (TCS)

TCS scores indicate the implementation level of comprehensive national tobacco control policies based on six cost-effective measures that should be prioritised according to the World Bank, including price, smoke-free laws, public spending on tobacco control, advertising bans, health warning, and treatment. This score increases with the strength of tobacco control policies up to a possible maximum of 100 points, indicating full implementation.^21^ For each EU MS, the most recent score available before each survey wave was used as an indicator of tobacco control policy implementation, accounting for lagged policy effects.

### Statistical analysis

Weights provided in the Eurobarometer datasets were applied to account for the sampling design when estimating prevalence of product use across the EU. Descriptive results are presented as percentages with 95% confidence intervals (CIs). Observations with missing values or responses of “Don’t know” or “Refusal” were excluded (n = 1,024; 4.8% of observations) (Supplementary Fig. S1).

We used the survey wave (year; hereafter wave) as a proxy for time to examine trends over time. Modified Poisson regression was used to estimate adjusted prevalence ratios (aPRs). Two multilevel Poisson regression models (*mepoisson*) with robust standard errors were used to examine associations between wave and ever and current use of waterpipe and e-cigarettes. Given the limited number of survey waves, wave was modelled as a categorical variable rather than assuming a linear trend or using joinpoint regression. These regression analyses were fitted without survey weights, because the variables used to construct the weights were included as covariates in the models, an approach supported in the survey methodology literature.^22^ Given the repeated cross-sectional design, multilevel models were specified with countries set as the higher-level unit, and relevant covariates were included to account for between-country and between-wave heterogeneity.

Initial models included age and gender as covariates based on established literature.^10^^,^^23^ Additional individual-level candidate confounders were assessed comparing alternative model specifications using Akaike Information Criterion and Bayesian Information Criterion to determine their contribution to model fit and parsimony. After individual-level covariates had been finalised, country-level variables were incorporated. Final models were adjusted for area of residence, financial circumstance, smoking status, AIC per capita and TCS score. Analyses of current use were restricted to data from 2017 to 2023. To examine changes over time, we treated wave as a categorical variable with 2017 as the reference year, representing the midpoint of the study period and the first year with comparable data available on current use for waterpipe.

To explore whether trends differed between sociodemographic characteristics, interaction terms between each sociodemographic variable and wave were tested for statistical significance. To account for multiple testing across these interaction terms, p-values were adjusted using the Benjamini–Hochberg false discovery rate procedure.^24^ Statistically significant interactions after correction were used to define primary stratified analysis, with models stratified by the relevant sociodemographic factor while adjusting for other covariates. For sociodemographic variables whose interaction with wave did not remain statistically significant after correction in a given model, stratified results are presented as exploratory. This allowed examination of temporal changes in product use across subgroups.

Sensitivity analyses were conducted using alternative definitions of ever and current use of waterpipe and e-cigarette, including ever regular use (≥occasionally; respondents reporting having tried it once or twice were classified as never users), ever use (experimentation; respondents reporting ever regular use were set to missing), current use including less-than-monthly use, and current daily use, each compared with never use. To assess the potential impact of methodological deviations in the 2020 survey wave, all primary regression analyses for ever and current use of waterpipe and e-cigarettes were repeated after excluding the 2020 wave, and estimates were compared with the main analyses to evaluate the robustness of observed temporal trends.

Results are presented as aPRs with 95% CI, with statistical significance set at p-value < 0.05. All statistical analyses were performed using Stata V19.5.

### Role of the funding source

The funders did not influence the study design, data collection, data analysis, interpretation, or writing of the manuscript.

### Ethics statement

This study involved a secondary analysis of publicly available, fully anonymised data and therefore did not require ethical approval. The requirement for written informed consent was waived because the analysis used anonymised data with no possibility of participant identification.

## Results

**Fig. 1:**
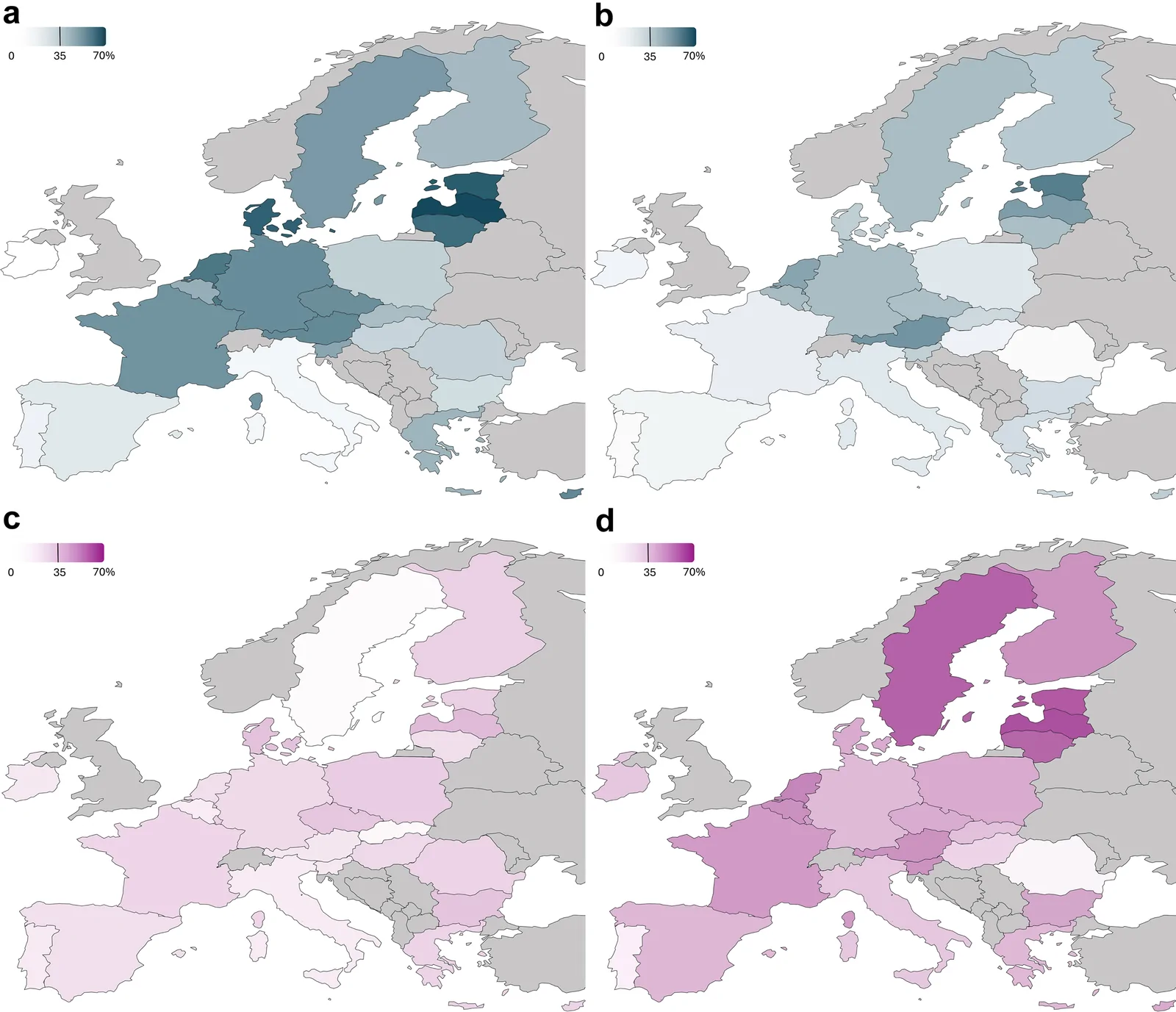
**Prevalence of ever waterpipe and e-cigarette use among young people across European Union Member States in 2012 and 2023.** (a) Prevalence of ever waterpipe use (2012). (b) Prevalence of ever waterpipe use (2023). (c) Prevalence of ever e-cigarette use (2012). (d) Prevalence of ever e-cigarette use (2023). Regions with no data or excluded from the analyses are coloured in grey.

The prevalence of ever use of waterpipe and e-cigarettes varied widely across EU MS (Fig. 1). For waterpipe, prevalence was 31.2% (95% CI: 29.1–33.3%) in 2012, 35.0% (32.8–37.4%) in 2014, 29.1% (26.8–31.4%) in 2017, 31.8% (29.6–34.2%) in 2020, and 14.9% (13.2–16.8%) in 2023. Among minors, who are not legally permitted to purchase tobacco- or nicotine-containing products, prevalence declined from 25.2% in 2012 to 4.0% in 2023 (Table 1). Country-level prevalence ranged from 1.7% in Portugal (2023) to 75.5% in Sweden (2014). The greatest reduction between 2012 and 2023 was observed in Denmark (−42.2%), whereas Italy recorded minimal change (−0.1%) (Supplementary Table S4).

**Table 1:** Adjusted prevalence ratios for ever-use of waterpipe and e-cigarettes among individuals aged 15–30 years in the European Union (2012–23), by sociodemographic group.

	2012		2014		2017	2020		2023	
	Weighted prevalence (%)	aPRs (95% CI)	Weighted prevalence (%)	aPRs (95% CI)	Weighted prevalence (%)	Weighted prevalence (%)	aPRs (95% CI)	Weighted prevalence (%)	aPRs (95% CI)
**Ever use of waterpipe**									
Gender					REF. Year				
Woman	25.7%	1.10 (0.97–1.24)	30.1%	**1.15 (1.01–1.30)**	23.1%	27.7%	**1.24 (1.02–1.52)**	12.9%	**0.51 (0.41–0.64)**
Man	36.3%	1.04 (0.94–1.15)	39.6%	1.09 (0.98–1.21)	34.7%	35.7%	1.08 (0.90–1.30)	16.9%	**0.56 (0.44–0.72)**
Age (years)									
15–17	25.2%	**1.39 (1.07–1.81)**	24.2%	**1.36 (1.07–1.73)**	19.0%	14.5%	1.02 (0.71–1.46)	4.0%	**0.44 (0.29–0.68)**
18–24	35.3%	1.05 (0.95–1.17)	38.9%	**1.15 (1.01–1.32)**	31.7%	32.1%	1.10 (0.90–1.34)	16.8%	**0.56 (0.44–0.70)**
25–30	27.9%	1.00 (0.89–1.13)	33.6%	1.03 (0.94–1.13)	28.6%	36.5%	**1.25 (1.06–1.49)**	15.7%	**0.55 (0.44–0.70)**
Area of residence (*exploratory*)									
Rural	25.2%	0.93 (0.77–1.12)	33.5%	1.12 (0.93–1.33)	27.2%	26.3%	1.13 (0.97–1.32)	9.7%	**0.53 (0.40–0.70)**
Urban	33.9%	**1.12 (1.02–1.23)**	35.6%	**1.12 (1.02–1.24)**	29.8%	33.9%	1.18 (0.96–1.44)	16.8%	**0.54 (0.43–0.68)**
Difficulty paying bills									
Almost Never/Never	33.2%	1.06 (0.94–1.18)	35.7%	1.11 (0.98–1.25)	30.8%	31.3%	1.06 (0.89–1.26)	12.8%	**0.46 (0.37–0.57)**
Most of the Time/ From Time to Time	28.3%	1.08 (0.94–1.24)	34.0%	1.12 (0.98–1.28)	26.3%	33.0%	**1.36 (1.09–1.70)**	18.8%	**0.71 (0.54–0.93)**
**Ever use of E-cigarettes**									
Gender									
Woman	9.8%	**0.46 (0.39–0.56)**	15.9%	**0.76 (0.65–0.88)**	19.1%	20.1%	**1.20 (1.01–1.43)**	25.5%	**1.41 (1.18–1.69)**
Man	14.5%	**0.48 (0.41–0.56)**	21.8%	**0.76 (0.67–0.86)**	28.0%	26.7%	1.11 (0.99–1.25)	26.2%	1.11 (0.95–1.29)
Age (years)									
15–17	8.6%	**0.44 (0.32–0.61)**	12.7%	0.77 (0.57–1.03)	18.3%	16.6%	1.02 (0.80–1.29)	16.5%	1.28 (0.93–1.75)
18–24	13.0%	**0.47 (0.39–0.56)**	19.8%	**0.74 (0.63–0.87)**	25.9%	24.5%	1.15 (0.99–1.33)	31.5%	**1.28 (1.12–1.46)**
25–30	12.4%	**0.48 (0.41–0.57)**	19.9%	**0.77 (0.69–0.86)**	22.5%	24.4%	**1.17 (1.02–1.34)**	21.4%	1.17 (0.96–1.42)
Area of residence (*exploratory*)									
Rural	11.4%	**0.40 (0.32–0.50)**	18.6%	**0.69 (0.59–0.80)**	24.5%	21.4%	0.97 (0.82–1.14)	24.0%	1.17 (0.94–1.46)
Urban	12.6%	**0.50 (0.43–0.59)**	19.1%	**0.78 (0.68–0.89)**	23.3%	24.3%	**1.23 (1.09–1.39)**	26.5%	**1.27 (1.11–1.46)**
Difficulty paying bills									
Almost Never/Never	12.7%	**0.50 (0.43–0.59)**	17.1%	**0.76 (0.66–0.87)**	22.4%	21.7%	**1.16 (1.03–1.31)**	25.1%	**1.38 (1.20–1.60)**
Most of the Time/ From Time to Time	11.5%	**0.44 (0.36–0.54)**	22.2%	**0.75 (0.65–0.88)**	25.7%	27.3%	1.15 (0.99–1.34)	27.2%	1.06 (0.89–1.27)

Data are adjusted prevalence ratios (aPRs) with 95% CIs. Models were stratified by each sociodemographic factor (gender, age, area of residence, difficulty in paying bills), and adjusted for smoking status, Actual Individual Consumption, Tobacco Control Scale score, and all other listed covariates. The 2017 survey wave serves as the reference category. Values in **bold** indicate statistical significance at p < 0.05. aPR, adjusted prevalence ratio; CI: Confidence interval.

Prevalence of ever e-cigarette use increased from 12.2% (10.8–13.8%) in 2012 to 25.8% (23.7–28.2%) in 2023 (Fig. 1). Among 15–17-year-olds, a similar upward pattern was observed, with prevalence raising from 8.6% in 2012 to 16.5% in 2023 (Table 1). Country-level prevalence ranged from 1.2% (0.3–12.7%) in Sweden (2012) to 58.5% (49.8–66.8%) in Latvia (2023). Most countries observed increases, ranging from 0.9% in Hungary to 50.8% in Sweden. Only Portugal and Romania recorded declines of 2.0% and 10.0%, respectively (Supplementary Table S5).

**Fig. 2:**
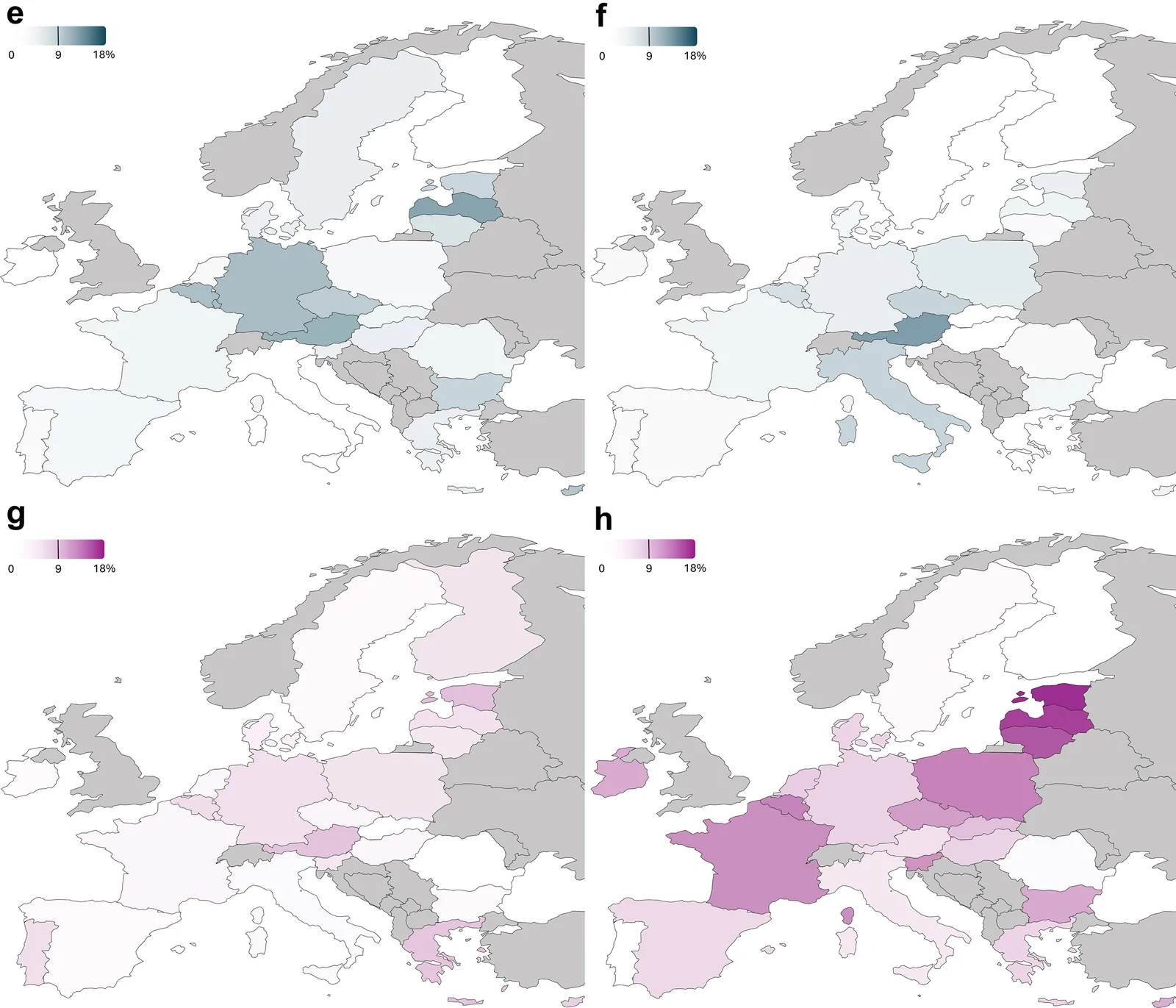
**Prevalence of current waterpipe and e-cigarette use among young people across European Union Member States in 2017 and 2023.** (e) Prevalence of current waterpipe use (2017). (f) Prevalence of current waterpipe use (2023). (g) Prevalence of current e-cigarette use (2017). (h) Prevalence of current e-cigarette use (2023). Regions with no data or excluded from the analyses are coloured in grey.

Prevalence of current waterpipe use was 2.5% (1.8–3.5%) in 2017, 5.1% (4.1–6.4%) in 2020, and 1.8% (1.2–2.7%) in 2023 (Fig. 2). Among minors, prevalence declined from 5.4% to 0.1% over the same period (Table 2). Estimates ranged from 0.4% in Finland (2020) and the Netherlands (2023) to 18.5% in Luxembourg (2020). France and Portugal recorded minimal decreases (<0.1%) while Latvia had the greatest decline (−7.5%) from 2017 to 2023. In contrast, Poland and Austria observed increases of 1.3% and 2.1%, respectively (Supplementary Table S6).

Prevalence of current e-cigarette use increased across EU, rising from 1.6% (1.1–2.3%) in 2017 to 5.3% (4.0–6.8%) in 2023 (Fig. 2). Similarly, among 15–17-year-olds, prevalence rose from 1.2% to 4.4% (Table 2). Country-level prevalence ranged from 0.4% in Ireland and Spain (2017) to 18.0% in Estonia (2023). Latvia recorded the largest increase in prevalence (+13.8%), whereas Sweden showed the smallest change (<0.1%). Declines were observed only in Austria (−2.5%) and Greece (−1.3%) (Supplementary Table S7). Prevalence could not be estimated for several countries for either product due to very low estimates or lack of observations.

**Table 2:** Adjusted prevalence ratios for current-use of waterpipe and e-cigarettes among individuals aged 15–30 years in the European Union (2017–23), by sociodemographic group.

	2017	2020		2023	
	Weighted prevalence (%)	Weighted prevalence (%)	aPRs (95% CI)	Weighted prevalence (%)	aPRs (95% CI)
**Current use of waterpipe**					
Gender	REF. Year				
Woman	1.6%	3.3%	**2.30 (1.46–3.63)**	2.0%	1.19 (0.57–2.47)
Man	3.4%	6.7%	**1.85 (1.18–2.90)**	1.6%	**0.47 (0.31–0.72)**
Age (years)					
15–17	5.4%	4.3%	1.50 (0.77–2.93)	0.1%	**0.08 (0.01–0.76)**
18–24	2.5%	5.7%	**2.16 (1.29–3.64)**	2.2%	0.57 (0.30–1.07)
25–30	1.8%	4.6%	**1.92 (1.14–3.22)**	1.7%	1.00 (0.54–1.84)
Area of residence					
Rural	2.1%	3.9%	1.63 (0.96–2.78)	0.3%	**0.23 (0.11–0.48)**
Urban	2.6%	5.5%	**2.07 (1.39–3.07)**	2.3%	0.82 (0.49–1.40)
Difficulty paying bills					
Almost Never/Never	2.9%	4.2%	**1.57 (1.01–2.44)**	1.1%	0.60 (0.28–1.30)
Most of the Time/ From Time to Time	1.9%	6.9%	**2.76 (1.66–4.60)**	3.0%	0.81 (0.41–1.63)
**Current use of E-cigarettes**					
Gender					
Woman	0.7%	1.7%	**2.12 (1.15–3.91)**	6.6%	**8.47 (4.57–15.71)**
Man	2.4%	3.9%	1.41 (1.00–1.97)	4.0%	**2.52 (1.63–3.94)**
Age (years) (*exploratory*)					
15–17	1.2%	2.6%	1.54 (0.64–3.68)	4.4%	**4.48 (2.30–8.74)**
18–24	1.4%	3.2%	**1.64 (1.05–2.58)**	6.2%	**4.58 (2.67–7.83)**
25–30	1.9%	2.5%	1.52 (0.94–2.44)	4.3%	**3.64 (2.15–6.17)**
Area of residence (*exploratory*)					
Rural	1.6%	3.3%	1.48 (0.76–2.88)	5.9%	**3.29 (1.82–5.94)**
Urban	1.6%	2.7%	**1.75 (1.22–2.49)**	5.0%	**4.82 (2.91–7.99)**
Difficulty paying bills (*exploratory*)					
Almost Never/Never	1.4%	2.5%	1.30 (0.89–1.90)	4.7%	**4.38 (3.11–6.18)**
Most of the Time/ From Time to Time	1.8%	3.6%	**2.00 (1.15–3.47)**	6.4%	**3.61 (1.66–7.86)**

Data are adjusted prevalence ratios (aPRs) with 95% CIs. Models were stratified by each sociodemographic factor (gender, age, area of residence, difficulty in paying bills), and adjusted for smoking status, Actual Individual Consumption, Tobacco Control Scale score, and all other listed covariates. Values in **bold** indicate statistical significance at p < 0.05. The 2017 survey wave serves as the reference category. aPR, adjusted prevalence ratio; CI, Confidence interval.

**Fig. 3:**
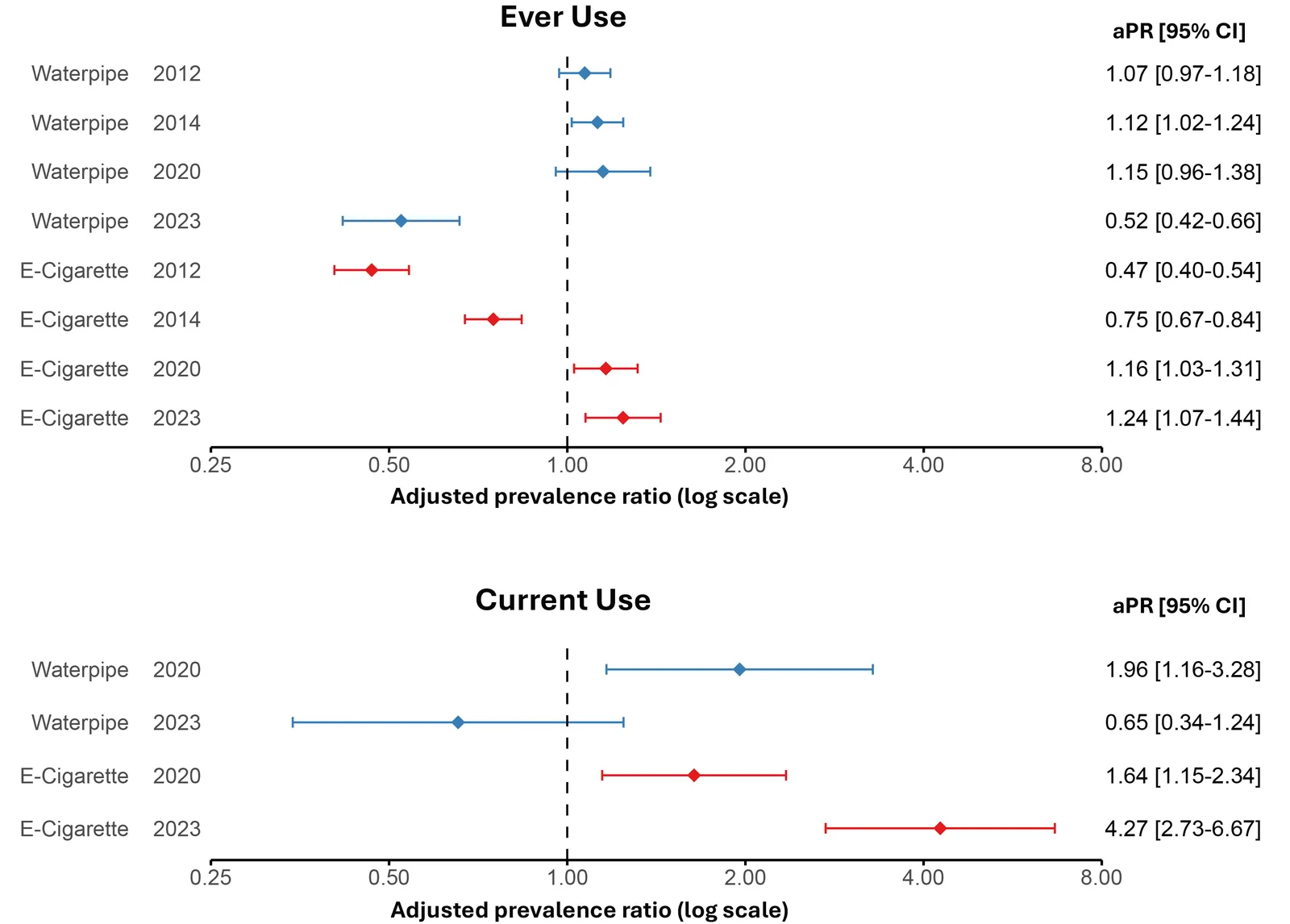
**Adjusted prevalence ratios for prevalence of ever and current waterpipe and e-cigarette use across waves relative to 2017.** aPR: Adjusted prevalence ratio. CI: Confidence interval.

The Poisson regression models showed that, compared with 2017, ever waterpipe use was significantly lower in 2023 (aPR: 0.52, 0.42–0.66) and higher in 2014 (aPR: 1.12, 1.02–1.24), while e-cigarette use was significantly lower in pre-2017 waves (2012 aPR: 0.47, 0.40–0.54; 2014 aPR: 0.75, 0.67–0.84), and higher in 2020 (aPR: 1.16, 1.03–1.31) and in 2023 (aPR: 1.24, 1.07–1.44). Current waterpipe use was higher in 2020 (aPR: 1.96, 1.16–3.28) relative to 2017, while e-cigarette use increased in both 2020 (aPR: 1.64, 1.15–2.34) and 2023 (aPR: 4.27, 2.73–6.67) (Fig. 3).

Trends in ever waterpipe use varied across sociodemographic subgroups in earlier waves (Table 1). Among women, prevalence decreased in 2017 compared with 2014 (aPR: 1.15, 1.01–1.30) and increased again in 2020 (aPR: 1.24, 1.02–1.52). By age, higher prevalence relative to 2017 occurred earlier among younger groups and later among older groups: increases were observed in 2012 and 2014 among 15–17-year-olds, in 2014 among 18–24-year-olds, and in 2020 among 25–30-year-olds. Changes over time were broadly similar by financial circumstances, except in 2020, when prevalence increased among individuals reporting financial difficulty compared with 2017 (aPR: 1.36, 1.09–1.70). In 2023, all subgroups showed a statistically significant decline in prevalence relative to 2017 (aPR range: 0.44–0.71).

Across all sociodemographic subgroups, the prevalence of ever e-cigarette use was significantly lower in 2012 and 2014 than in 2017 (aPR range: 0.40–0.78) (Table 1). Post-2017 trends differed across groups, with women, urban residents, and those without financial difficulties observing greater relative increases in prevalence than their counterparts in 2020 and 2023. By age, an increase was observed in 2020 among 25–30-year-olds (aPR: 1.17, 1.02–1.34) and in 2023 among 18–24-year-olds (aPR: 1.28, 1.12–1.46), but not among other age groups.

Most subgroups recorded higher prevalence of current waterpipe use in 2020 relative to 2017, with varied magnitudes (Table 2). Relative increases were observed among both genders, those with and without financial difficulties, 18–24- and 25–30-year-olds, and urban residents. By 2023, statistically significant declines were observed among men, the youngest subgroup, and rural residents, but not among their counterparts.

Between 2017 and 2023, current e-cigarette use increased substantially across all subgroups (aPR range: 2.52–8.47) (Table 2). Among women, prevalence doubled in 2020 (aPR: 2.12, 1.15–3.91) and increased eightfold by 2023 (aPR: 8.47, 4.57–15.71), whereas among men prevalence increased 2.52-fold in 2023 (95% CI: 1.63–3.94).

Sensitivity analyses yielded estimates consistent in direction and magnitude with the primary analyses for both products at the EU level, with minor variations across alternative definitions and after exclusion of the 2020 wave; some estimates, particularly for waterpipe current use, lost statistical significance, whereas increasing trends in e-cigarette use remained robust (Supplementary Tables S8–S10).

## Discussion

Substantial variation was observed in the prevalence of ever and current use of waterpipe and e-cigarettes across EU MS, with current use remaining low despite higher levels of ever use. Our study shows contrasting temporal patterns in waterpipe and e-cigarette use (2012–2023): waterpipe use declined from 2017, with a temporary peak in 2020, whereas e-cigarette use increased substantially across most population groups throughout the study period. Trends in ever and current waterpipe use differed primarily by age group, with more pronounced variation across cohorts over time. By contrast, trends by gender and financial circumstance were less distinct, generally following similar trajectories over time, differing mainly in magnitude rather than direction. For e-cigarettes, increases in ever and current use were more pronounced among women, who showed the largest relative increases over time.

The observed levels of use among young people exceed estimates in EU-wide analyses of the general adult population for both products,^16^, ^17^, ^18^ highlighting the need for targeted prevention strategies, given the long-term risk of nicotine dependence with early exposure.^8^ However, prevention approaches may require different strategies for experimentation versus more sustained patterns of use. The lower prevalence of current use compared with ever use, suggests that experimentation remains common among young people, consistent with previous EU analysis.^17^ Curiosity and flavour appeal are commonly reported motivations for experimentation with both waterpipe and e-cigarettes,^25^^,^^26^ whereas regular e-cigarette use is more frequently associated with smoking reduction or cessation attempts.^25^ A qualitative study also suggests greater social acceptability of experimental e-cigarette use, whereas more regular use is less accepted,^27^ and waterpipe use remains strongly embedded in social contexts, which may limit progression to habitual use. A concerning pattern is co-use or transition to smoking,^14^^,^^16^ which has been linked to underestimation of nicotine dependence and the perception that occasional use is non-harmful.^28^ The role of e-cigarettes and other novel nicotine products in tobacco harm reduction remains the subject of ongoing debated; however, the present study was not designed to evaluate this framework. Rather, it provides descriptive evidence on population-level trends in ever and current e-cigarette use to inform tobacco control policy. These findings underscore the importance of distinguishing determinants of experimentation from those associated with regular use.

Furthermore, to assess whether our findings were sensitive to how ever and current use were defined, we explored alternative indicators, distinguishing between ever regular use, ever use (limited to experimentation), redefining current use to include those using less than monthly, and incorporating daily use as a marker of intensity. These additional analyses yielded broadly consistent temporal patterns, suggesting that our conclusions about changing experimentation and recent use among young people are unlikely to be materially affected by the specific operational definitions of ever and current use.

The concurrent decline in waterpipe use and increase in e-cigarette use among this age group mirrors patterns observed in the wider adult EU population.^2^^,^^29^ E-cigarettes may be more accessible at the population level, with fewer logistical and financial barriers compared with waterpipe use, which is often restricted to hospitality venues and requires greater coordination and costs. Evidence from a German cohort suggests that waterpipe use may precede later e-cigarette initiation among adolescents,^30^ which may be consistent with overlap in patterns of product ever use. Patterns across Northern European countries in this study are compatible with this hypothesis, with Estonia, Latvia, and Lithuania showing co-occurring high prevalence of both products. However, this represents an ecological inference based on country-level co-occurrence; these ecological patterns do not provide evidence of individual-level transitions between products, and no causal relationship or directional substitution can be inferred. Given that waterpipe use typically occurs in social settings, this may reflect contexts in which experimentation with nicotine-containing products co-occurs. These patterns may also be consistent with shared liability to nicotine use behaviours, whereby common genetic, psychological, and social factors predispose individuals to experiment with multiple products rather than reflecting directional transitions between them.^31^ Evidence from an ecological study of tobacco control policies in Europe and Canada found that policies targeting social norms around smoking were most strongly associated with declines in adolescent smoking,^32^ highlighting the importance of addressing social context in prevention strategies.

We observed aged-related heterogeneity in waterpipe use, with successive waves showing higher lifetime exposure in older cohorts, whereas exposure to e-cigarettes increased across all age groups. These patterns suggest age- and cohort-related differences in alternative tobacco and nicotine-containing product use among young people, although no directional substitution between products can be inferred. Nonetheless, the combination of declining waterpipe use and rising e-cigarette use is consistent with population-level changes in patterns of product use over time, whereby youth nicotine experimentation may be shifting from an analogue, venue-based social activity (waterpipe) towards a more digital, highly individualised, and, in some context, more affordable activity (e-cigarettes), in line with evolving product design and marketing strategies. Our findings therefore are compatible with the interpretation that waterpipes and e-cigarettes may play overlapping roles as youth experimentation products at the population level, without implying that one product has directly replaced the other or that they are equivalent in terms of health risk.

Financial circumstances shaped prevalence of use differently for each product. Young people reporting financial difficulties showed greater increases in waterpipe use in 2020, despite COVID-19-related social restrictions, which would have been expected to reduce use. Shifts in waterpipe use from public venues to homes during COVID-19 restrictions may have coincided with increased opportunities for regular use, especially in households with existing waterpipe users.^26^ However, this finding should be interpreted with caution given the methodological deviations affecting that wave.

For e-cigarettes, exposure increased among those with minimal financial difficulties. Individuals with greater spending capacity may be more likely to experiment, while e-cigarettes may serve as a lower-cost alternative for reducing cigarette consumption among some individuals with fewer resources.^25^ Economic considerations may thus influence ever use among price-sensitive young people, as demonstrated previously, where higher cigarette prices are linked to increased e-cigarette use.^33^ The absence of harmonised e-cigarette taxation across the EU creates price differentials between MS and facilitates cross-border purchases, and could be addressed by including e-cigarettes within the EU Tobacco Taxation Directive.^34^

A prominent finding was the nearly eightfold increase in current e-cigarette use among women. While earlier literature has found higher prevalence among men,^10^ the observed patterns may reflect staggered adoption across genders. This warrants attention, as it suggests that women represent an increasingly affected group, consistent with potential influences such as gendered digital marketing practices. However, it is important to note that e-cigarette use may reflect heterogeneous behavioural contexts, including experimentation among people who have never smoked as well as use among individuals attempting to reduce or quit smoking. User-led lifestyle branding may circumvent existing marketing restrictions under the Tobacco Products Directive (TPD),^35^ and is enabled by regulatory fragmentation across the Unfair Commercial Practices Directive,^36^ the Digital Services Act,^37^ and the Audiovisual Media Services Directive,^38^ none of which provides comprehensive coverage of user-based promotion of nicotine-containing products. Furthermore, the TPD’s failure to regulate the visual device design, unlike traditional cigarettes or flavour appeal, leaves a critical gap that may be associated with regular usage. This surge in regular e-cigarette use, alongside patterns observed for waterpipes, is consistent with population-level continuity in nicotine use behaviours rather than demonstrating individual-level initiation or transition between products in this group. Consequently, gender-specific trajectories of use and associated health outcomes must be monitored, with targeted policy interventions considered to address the appeal and promotion of e-cigarettes among young women.

To our knowledge, this is the first study to examine trends in waterpipe and e-cigarette use among young people in the EU overall and by sociodemographic characteristics. This approach addresses a previously underexplored research question by examining these products in parallel. Stratification by sociodemographic factors enabled identification of distinct trends between population subgroups to inform targeted interventions and research.

As a repeated cross-sectional design, the study does not allow inference on individual-level transitions between products, and all observed patterns reflect population-level prevalence and co-occurrence rather than behavioural trajectories. Accordingly, it cannot provide evidence that rising e-cigarette use has directly caused a decline in waterpipe use. The categorisation of smoking status (current, former, never) does not fully capture smoking uptake, intensity, duration, or patterns of multiple product use. In the absence of more detailed smoking histories, these patterns cannot distinguish between initiation, dual or poly-use, or substitution, and should therefore be interpreted as population-level prevalence trends rather than evidence of individual-level transition pathways. In addition, the findings reflect the 27 current EU Member States, and therefore may not be generalisable to countries with different regulatory approaches.

Additionally, small sample sizes in subgroup analyses for current use and in certain countries introduced uncertainty, reflected in wider CIs. Readers should therefore interpret these subgroup estimates with caution. This was compounded by methodological deviations in 2020, which may have affected comparability with other waves. We conducted sensitivity analyses excluding the 2020 wave, presented in the Supplementary Material (Supplementary Tables S9 and S10). Without the 2020 wave, no spike in current waterpipe use was observed. Given that this spike may reflect methodological factors and/or the unique circumstances of the COVID-19 pandemic, it is uncertain whether it should be considered in the interpretation of longer-term trends. Nonetheless, the main trends reported are based on comparisons between 2012/2017 and 2023, when the methodology was consistent.

Observations with missing data were excluded (4.8% of the analytical sample). Missingness was assumed to be broadly consistent with a Missing at Random mechanism, although this cannot be formally verified. While any deviation from this assumption could introduce bias, the relatively small proportion of missing data suggests that any resulting bias is likely to be limited, though it remains a study limitation. Moreover, weighting was applied to the analyses, ensuring that the results are representative of young people in the EU.

Self-reported measures of product use are widely used and well validated in epidemiological research, particularly in confidential survey settings such as those used in this study.^39^^,^^40^ While some degree of reporting error and misclassification cannot be excluded, this is unlikely to be systematic across countries or sociodemographic groups or to substantially affect the observed associations. Additionally, variations in question phrasing, structure, and response categories across waves may have introduced some reporting variability, potentially affecting comparability between waves.^41^ However, these differences were conceptually minor, as key measures such as ever use consistently captured whether an individual had ever tried or used the product. As such, any resulting variability is likely small and unlikely to materially influence the observed trends.

In conclusion, there was an overall decrease in waterpipe use and an increase in e-cigarette use between 2012 and 2023. Fewer young people are now exposed to waterpipe in the EU, whereas more are exposed to e-cigarettes. These findings suggest that, although waterpipes and e-cigarettes differ in composition, risk profile, and role in adult harm-reduction frameworks, they may play overlapping roles as vehicles for nicotine experimentation and regular use among young people at the population level. They also suggest the need for gender- and price-targeted strategies to reduce nicotine use among young people in the EU and support the goal of a tobacco-free generation. Ongoing surveillance is needed to capture evolving patterns of nicotine use and changes in the distribution of risk across subgroups. Insights into patterns of use, such as social settings, reasons for initiation, and continuation, would provide valuable context for profiling user groups and tailoring interventions.

## Contributors

MST, FTF and AF conceived the study. MST led the data analysis and prepared the initial draft, with input from FTF and AF. All authors (MST, FTF, CXL, AAL, EF, CM, AP, CIV, and AF) contributed to data interpretation and reviewed and edited the manuscript. FTF and AF had full access to and verified all the data used in this analysis and are the guarantors of the data. FTF and AF had final responsibility for the decision to submit the manuscript for publication.

## Data sharing statement

Eurobarometer datasets are owned by the European Commission. Data may be obtained from a third party and are publicly available (https://search.gesis.org/research_data/).

## Editor note

The Lancet Group takes a neutral position with respect to territorial claims in published maps and institutional affiliations.

## Declaration of interests

All authors declare no competing interests. The funder did not influence the results/outcomes of the study despite author affiliations with the funder.
