# Supplementary material for: Trends in waterpipe and e-cigarette use among young people in the European Union, 2012–2023: a repeated cross-sectional study
Source: Lancet Reg Health Eur. 2026 Aug 13;69:101815. doi: 10.1016/j.lanepe.2026.101815 (PMC13499096; doi:10.1016/j.lanepe.2026.101815)
Supplement: Supplementary file 1 [file mmc1.docx]

**Supplementary Materials**

[Figure S1: Sample construction flowchart	2](#_Toc221824880)

Table S1: Operationalisation of waterpipe and e‑cigarette ever use across Eurobarometer waves (2012–2023)	3

[Table S2: Wave and sociodemographic characteristics of study sample (N=20,466)](#_Toc221824880)	4

[Table S3: Number of observations and weighted percentages from each EU MS (N = 20,466)](#_Toc221824881)	5

[Table S4: Prevalence of ever waterpipe use in each EU MS across waves](#_Toc221824882)	6

[Table S5: Prevalence of ever e-cigarette use in each EU MS across waves](#_Toc221824883)	7

[Table S6: Prevalence of current waterpipe use in each EU MS across waves](#_Toc221824884)	8

[Table S7: Prevalence of current e-cigarette use in each EU MS across waves](#_Toc221824885)	9

Table S8: Adjusted prevalence ratios (aPRs) of alternative definitions of ever and current use of waterpipe among individuals aged 15–30 years in the European Union (2012–23).	10

Table S9: Adjusted prevalence ratios (aPRs) for ever-use of waterpipe and e-cigarettes among individuals aged 15–30 years in the European Union (2017–23), by sociodemographic group, after excluding 2020 data	11

Table S10: Adjusted prevalence ratios (aPRs) for current-use of waterpipe and e-cigarettes among individuals aged 15–30 years in the European Union (2017–23), by sociodemographic group, after excluding 2020 data	12

**[Figure S1:](#_Toc221824880)** [Sample construction flowchart](#_Toc221824880)

**Table S1: Operationalisation of waterpipe and e cigarette ever use across Eurobarometer waves (2012–2023)**

| **Wave** | **Question** | **Response options** | **Classification** |
| --- | --- | --- | --- |
| **Waterpipe** | | | |
| **2012 2014** | Have you ever tried any of the following products? 1. Water pipe (shisha, hookah) | 1. Yes, you use or used it regularly 2. Yes, you use or used it occasionally 3. Yes, you tried it once or twice 4. No | Ever use (1-3)  Never use (4) |
| **2017 2020** | Have you ever used or tried any of the following products? 1. Water pipe (shisha, hookah) | 1. Yes, every day 2. Yes, every week 3. Yes, every month 4. Yes, less than monthly 5. Yes, occasionally or regularly but you stopped 6. Yes, but only once or twice 7. Never | Ever use (1-6)  Never use (7) |
| **2023** | How often do you use the following products? 3. Water pipe tobacco (shisha, hookah) | 1. Every day 2. Every week 3. Every month 4. Less than monthly 5. You used to use it regularly, but you have stopped 6. You have tried it only once or twice 7. Never | Ever use (1-6)  Never use (7) |
| **E-cigarettes** | | | |
| **2012** | Have you ever tried any of the following products? 3. Electronic cigarettes | 1. Yes, you use or used it regularly 2. Yes, you use or used it occasionally 3. Yes, you tried it once or twice 4. No | Ever use (1-3)  Never use (4) |
| **2014** | Regarding the use of electronic cigarettes or any similar electronic devices (e-shisha, e-pipe), which of the following statements applies to you? | 1. You currently use electronic cigarettes or similar electronic devices (e.g. e-shisha, e-pipe) 2. You used them in the past, but no longer use them 3. You tried them in the past but no longer use them 4. You have never used them | Ever use (1-3)  Never use (4) |
| **2017** | Which of the following statements about the use of electronic cigarettes or any similar electronic devices  (e-shisha, e-pipe) applies to you? | 1. You currently use electronic cigarettes or similar electronic devices (e.g. e-shisha, e-pipe) 2. You used to use them, but no longer do so 3. You have tried them once or twice 4. You have never tried or used them | Ever use (1-3)  Never use (4) |
| **2020 2023** | Thinking about the following products, which of the following applies to you? 1. E-cigarettes | 1. You currently use it 2. You used to use it but you have stopped 3. You have tried it only once or twice 4. You have never used it | Ever use (1-3)  Never use (4) |

**Table S2: Wave and sociodemographic characteristics of study sample (N=20,466)**

| **Characteristics** | **N** | **Weighted Percentage (%)** |
| --- | --- | --- |
| **Wave** |  |  |
| 2012 | 4,553 | 20.4 |
| 2014 | 4,149 | 19.9 |
| 2017 | 3,892 | 19.6 |
| 2020 | 4,109 | 20.1 |
| 2023 | 3,763 | 20.1 |
| **Gender** |  |  |
| Man | 9,885 | 51.6 |
| Woman | 10,581 | 48.4 |
| **Age** (years) |  |  |
| 15-17 | 2,447 | 11.5 |
| 18-24 | 8,856 | 48.1 |
| 25-30 | 9,163 | 40.5 |
| **Area of Residence** |  |  |
| Rural | 5,934 | 27.4 |
| Urban | 14,532 | 72.6 |
| **Difficulty in Paying Bills** |  |  |
| Almost never/never | 12,179 | 63.1 |
| Most of the time/from time to time | 8,287 | 37.0 |

**Table S3: Number of Observations and Weighted Percentages from Each EU MS (N = 20,466)**

| **Country** | **N** | **Weighted Percentage (%)** |
| --- | --- | --- |
| Austria | 825 | 2.0 |
| Belgium | 984 | 2.3 |
| Bulgaria | 851 | 1.6 |
| Cyprus | 441 | 0.2 |
| Czech Republic | 912 | 2.5 |
| Denmark | 597 | 1.4 |
| Estonia | 875 | 0.3 |
| Finland | 583 | 1.3 |
| France | 880 | 15.2 |
| Germany | 1,199 | 18.5 |
| Greece | 926 | 2.7 |
| Hungary | 698 | 2.3 |
| Ireland | 859 | 1.2 |
| Italy | 779 | 11.2 |
| Latvia | 1,056 | 0.5 |
| Lithuania | 827 | 0.8 |
| Luxembourg | 416 | 0.1 |
| Malta | 303 | 0.1 |
| Netherlands | 633 | 3.9 |
| Poland | 887 | 9.9 |
| Portugal | 801 | 2.2 |
| Romania | 1,146 | 5.1 |
| Slovakia | 732 | 1.4 |
| Slovenia | 777 | 0.5 |
| Spain | 956 | 10.7 |
| Sweden | 523 | 2.0 |

**Table S4: Prevalence of Ever Waterpipe Use in Each EU MS across Waves**

| **Ever Use – Water pipe** | **Wave** | | | | | **Change**  **(2012-2023)** |
| --- | --- | --- | --- | --- | --- | --- |
|  | **2012** | **2014** | **2017** | **2020** | **2023** |  |
| **Country** | **% (95% CI)** | **% (95% CI)** | **% (95% CI)** | **% (95% CI)** | **% (95% CI)** | **%** |
| Austria | 47.2 (39.6 - 55.0) | 40.8 (32.5 - 49.8) | 45.1 (36.7 - 53.8) | 41.6 (33.9 - 49.8) | 41.3 (32.4 - 50.7) | -5.9 |
| Belgium | 34.1 (27.3 – 41.6) | 39.5 (32.0 - 47.5) | 37.4 (29.8 - 45.7) | 48.6 (39.7 - 57.6) | 26.2 (21.3 - 31.9) | -7.9 |
| Bulgaria | 17.6 (12.3 - 24.5) | 29.2 (23.0 - 36.4) | 24.1 (18.3 - 31.0) | 34.2 (27.5 - 41.7) | 13.3 (9.0 - 19.3) | -4.3 |
| Cyprus | 47.6 (39.6 – 55.7) | 49.2 (38.8 - 59.5) | 44.9 (33.2 - 57.2) | 52.4 (40.7 - 63.8) | 19.2 (10.9 - 31.5) | -28.4 |
| Czech Republic | 45.6 (38.4 - 53.0) | 44.1 (37.0 - 51.3) | 48.6 (41.0 - 56.2) | 34.4 (27.7 - 41.8) | 24.4 (17.2 - 33.3) | -21.2 |
| Denmark | 60.9 (53.0 - 68.2) | 60.8 (51.6 - 69.3) | 55.0 (45.1 - 64.6) | 51.2 (41.3 - 61.0) | 18.7 (11.7 - 28.7) | -42.2 |
| Estonia | 62.5 (55.5 - 69.0) | 56.5 (48.4 - 64.2) | 59.8 (50.0 - 69.0) | 63.9 (53.8 - 73.0) | 49.7 (42.8 - 56.5) | -12.8 |
| Finland | 30.1 (22.1 - 39.5) | 29.8 (22.9 - 37.8) | 30.4 (22.9 - 39.2) | 54.7 (32.3 - 75.4) | 20.6 (12.7 - 31.6) | -9.5 |
| France | 43.9 (36.6 - 51.5) | 54.9 (46.4 - 63.1) | 38.1 (30.3 - 46.5) | 32.0 (25.4 - 39.4) | 7.5 (4.3 - 12.9) | -36.4 |
| Germany | 46.0 (38.6 - 53.6) | 41.5 (35.0 - 48.2) | 38.9 (32.0 - 46.4) | 49.3 (42.5 - 56.1) | 25.1 (19.7 - 31.4) | -20.9 |
| Greece | 31.9 (26.0 - 38.4) | 15.6 (10.7 - 22.2) | 21.5 (15.9 - 28.5) | 14.5 (10.1 - 20.4) | 13.2 (8.9 - 19.2) | -18.7 |
| Hungary | 20.9 (15.2 - 27.9) | 26.8 (20.2 - 34.6) | 15.0 (9.7 - 22.3) | 16.7 (10.6 - 25.1) | 6.0 (2.7 - 12.7) | -14.9 |
| Ireland | 5.6 (3.2 - 9.8) | 8.3 (4.8 - 14.1) | 5.1 (2.5 - 10.0) | 41.9 (35.1 - 49.1) | 4.8 (2.5 - 9.2) | -0.8 |
| Italy | 9.3 (5.5 - 15.3) | 6.6 (3.4 - 12.4) | 7.7 (4.4 - 13.2) | 3.6 (1.6 – 7.7) | 9.2 (5.3 - 15.6) | -0.1 |
| Latvia | 69.7 (64.2 - 74.8) | 71.2 (64.2 - 77.4) | 75.2 (67.1 - 81.8) | 60.8 (53.3 - 67.8) | 38.3 (30.1 - 47.1) | -31.4 |
| Lithuania | 56.9 (50.5 - 63.0) | 67.3 (59.9 – 73.9) | 51.9 (43.2 - 60.6) | 47.0 (38.8 - 55.4) | 24.9 (17.9 - 33.4) | -32.0 |
| Luxembourg | 52.8 (41.9 - 63.3) | 44.6 (32.3 - 57.6) | 52.4 (40.3 - 64.2) | 67.7 (57.4 - 76.5) | 20.0 (11.8 - 31.9) | -32.8 |
| Malta | 17.3 (9.5 - 29.4) | 31.0 (20.9 - 43.3) | 19.3 (11.5 - 30.5) | 18.6 (9.5 - 33.3) | 11.9 (5.1 - 25.2) | -5.4 |
| Netherlands | 53.2 (45.5 - 60.8) | 45.5 (36.7 - 54.6) | 43.6 (34.6 - 53.1) | 36.6 (26.4 - 48.2) | 34.7 (25.1 - 45.9) | -18.5 |
| Poland | 22.2 (16.9 - 28.6) | 31.7 (25.6 - 38.5) | 14.3 (9.7 - 20.5) | 6.0 (3.0 - 11.9) | 9.5 (4.9 - 17.5) | -12.7 |
| Portugal | 10.7 (6.8 - 16.6) | 15.3 (10.1 - 22.4) | 6.0 (3.3 - 10.5) | 8.9 (5.3 - 14.5) | 1.7 (0.6 - 5.3) | -9.0 |
| Romania | 21.8 (16.8 - 27.8) | 20.7 (15.9 - 26.5) | 11.7 (8.0 - 16.7) | 11.2 (7.7 - 16.0) | 1.8 (0.7 - 4.7) | -20.0 |
| Slovakia | 28.8 (22.1 - 36.7) | 25.6 (19.0 - 33.4) | 31.0 (23.8 - 39.3) | 32.0 (25.1 - 39.7) | 15.2 (9.9 - 22.5) | -13.7 |
| Slovenia | 37.2 (30.6 - 44.3) | 35.4 (27.7 - 43.9) | 35.3 (27.6 - 44.0) | 32.6 (25.6 - 40.6) | 18.4 (12.4 - 26.4) | -18.8 |
| Spain | 14.1 (10.0 - 19.5) | 25.0 (19.4 - 31.5) | 29.2 (22.8 - 36.4) | 54.2 (46.4 - 61.9) | 5.1 (2.7 - 9.2) | -9.0 |
| Sweden | 41.9 (29.7 - 55.1) | 75.5 (64.1 - 84.1) | 61.8 (50.3 - 72.1) | 36.5 (29.9 - 43.7) | 25.3 (15.9 - 37.9) | -16.6 |
| **EU** | **31.2 (29.1 - 33.3)** | **35.0 (32.8 - 37.4)** | **29.1 (26.8 - 31.4)** | **31.8 (29.6 - 34.2)** | **14.9 (13.2 - 16.8)** | **-16.3** |

**Table S5: Prevalence of Ever E-cigarette Use in Each EU MS across Waves**

| **Ever Use –**  **E-cigarettes** | **Wave** | | | | | **Change (2012-2023)** |
| --- | --- | --- | --- | --- | --- | --- |
|  | **2012** | **2014** | **2017** | **2020** | **2023** |  |
| **Country** | **% (95% CI)** | **% (95% CI)** | **% (95% CI)** | **% (95% CI)** | **% (95% CI)** | **%** |
| Austria | 8.6 (5.3 - 13.8) | 16.4 (10.9 - 23.8) | 36.1 (28.1 - 45.0) | 26.7 (20.0 - 34.8) | 35.8 (27.5 - 44.9) | +27.2 |
| Belgium | 6.9 (3.9 - 12.1) | 18.3 (12.8 - 25.4) | 26.3 (19.9 - 34.0) | 37.2 (28.7 - 46.5) | 36.5 (31.0 - 42.4) | +29.6 |
| Bulgaria | 19.5 (14.0 - 26.5) | 19.7 (14.4 - 26.2) | 18.9 (13.8 - 25.5) | 17.2 (12.2 - 23.7) | 29.1 (22.7 - 36.4) | +9.6 |
| Cyprus | 14.5 (9.7 - 21.2) | 22.6 (15.0 - 32.7) | 33.7 (23.2 - 46.1) | 22.2 (14.1 - 33.2) | 27.6 (17.6 - 40.4) | +13.2 |
| Czech Republic | 18.7 (13.6 - 25.1) | 27.8 (21.6 - 34.9) | 38.0 (30.8 - 45.7) | 28.2 (22.1 - 35.3) | 26.8 (19.1 - 36.3) | +8.9 |
| Denmark | 20.5 (14.9 - 27.7) | 22.1 (15.5 - 30.5) | 26.6 (18.9 - 36.1) | 25.1 (17.4 - 34.9) | 27.4 (18.8 - 38.1) | +6.9 |
| Estonia | 16.2 (11.6 – 22.0) | 28.4 (21.7 – 36.1) | 47.3 (37.7 - 57.1) | 49.7 (40.7 - 58.6) | 54.8 (47.8 - 61.5) | +38.6 |
| Finland | 15.5 (9.8 - 23.7) | 32.4 (25.3 - 40.5) | 32.6 (25.0 - 41.4) | 23.7 (12.3 - 40.6) | 35.7 (25.2 - 47.8) | +20.2 |
| France | 13.5 (9.1 - 19.4) | 33.7 (26.2 - 42.2) | 32.9 (25.5 - 41.2) | 31.2 (24.7 - 38.6) | 33.0 (25.8 - 41.1) | +19.5 |
| Germany | 12.0 (8.0 - 17.7) | 16.2 (11.9- 21.6) | 26.7 (20.8 - 33.6) | 25.6 (20.1 - 31.9) | 22.8 (17.7 - 28.8) | +10.8 |
| Greece | 14.5 (10.5 - 19.7) | 7.7 (4.6 - 12.8) | 24.2 (18.1 - 31.4) | 21.0 (15.6 - 27.6) | 23.4 (17.6 - 30.4) | +8.9 |
| Hungary | 13.0 (8.6 - 19.2) | 11.7 (7.4 - 17.9) | 14.8 (9.6 - 22.2) | 17.2 (11.0 - 26.0) | 13.9 (8.6 - 21.6) | +0.9 |
| Ireland | 8.2 (5.1 - 13.1) | 14.3 (9.6 - 20.6) | 20.2 (14.4 - 27.6) | 46.6 (39.6 - 53.8) | 18.4 (12.8 - 25.8) | +10.2 |
| Italy | 7.3 (3.9 – 13.2) | 9.3 (5.6 - 15.2) | 11.9 (7.7 - 17.9) | 13.7 (9.3 - 19.7) | 18.3 (12.7 - 25.6) | +11.0 |
| Latvia | 22.3 (17.8 - 27.4) | 33.4 (26.7 - 40.8) | 51.6 (42.7 - 60.4) | 41.8 (34.5 - 49.4) | 58.5 (49.8 - 66.8) | +36.2 |
| Lithuania | 11.0 (7.7 - 15.7) | 21.0 (15.4 - 27.8) | 36.4 (28.2 - 45.5) | 40.4 (32.5 - 48.8) | 50.5 (41.8 - 59.2) | +39.5 |
| Luxembourg | 15.7 (9.3 - 25.4) | 26.8 (16.7 - 40.0) | 27.1 (17.7 - 39.1) | 35.0 (25.2 - 46.2) | 32.4 (21.2 - 45.9) | +16.7 |
| Malta | 6.9 (2.8 - 15.8) | 25.5 (16.3 - 37.6) | 14.8 (8.1 - 25.4) | 11.7 (4.9 - 25.4) | 18.9 (9.6 - 33.7) | +12.0 |
| Netherlands | 10.7 (6.9 - 16.3) | 15.4 (10.0 - 23.0) | 22.0 (15.3 - 30.7) | 21.2 (13.3 - 32.1) | 40.3 (29.9 - 51.7) | +29.6 |
| Poland | 17.1 (12.5 - 23.1) | 24.1 (18.7 - 30.5) | 21.5 (15.8 - 28.6) | 13.4 (8.5 - 20.6) | 27.4 (18.9 - 38.1) | +10.3 |
| Portugal | 7.7 (4.4 - 13.1) | 10.1 (6.0 - 16.7) | 17.6 (12.6 - 24.0) | 16.4 (11.4 - 23.1) | 5.7 (3.1 - 10.3) | -2.0 |
| Romania | 14.6 (10.5 – 20.1) | 15.3 (11.2 - 20.6) | 16.8 (12.4 - 22.2) | 13.5 (9.7 - 18.5) | 4.6 (2.5 - 8.3) | -10.0 |
| Slovakia | 3.2 (1.3 - 7.7) | 8.9 (5.2 - 14.7) | 18.6 (12.8 - 26.3) | 29.0 (22.3 - 36.7) | 20.9 (14.4 - 29.4) | +17.7 |
| Slovenia | 7.9 (4.8 - 12.8) | 14.3 (9.1 - 21.8) | 20.6 (14.4 - 28.6) | 21.3 (15.5 - 28.5) | 35.7 (27.7 - 44.7) | +27.8 |
| Spain | 10.3 (6.9 - 15.2) | 13.3 (9.1 - 18.9) | 20.0 (14.7 - 26.6) | 25.8 (19.6 - 33.1) | 23.6 (18.0 - 30.3) | +13.3 |
| Sweden | 1.2 (0.3 - 12.7) | 17.5 (10.5 - 27.7) | 17.1 (10.2 - 27.2) | 22.3 (16.9 - 29.0) | 52.0 (39.1 - 64.6) | +50.8 |
| **EU** | **12.2 (10.8 - 13.8)** | **19.0 (17.1 - 21.0)** | **23.6 (21.6 - 25.8)** | **23.5 (21.5 - 25.7)** | **25.8 (23.7 - 28.2)** | **+13.6** |

**Table S6: Prevalence of Current Waterpipe Use in Each EU MS across Waves**

| **Current Use –**  **Waterpipe** | **Wave** | | | **Change (2017-2023)** |
| --- | --- | --- | --- | --- |
|  | **2017** | **2020** | **2023** |  |
| **Country** | **% (95% CI)** | **% (95% CI)** | **% (95% CI)** | **%** |
| Austria | 7.6 (4.1 - 13.9) | 9.5 (5.9 - 14.9) | 9.7 (5.5 - 16.6) | +2.1 |
| Belgium | 6.3 (3.2 – 12.0) | 5.3 (2.5 - 10.9) | 3.2 (1.5 - 6.8) | -3.1 |
| Bulgaria | 4.2 (2.0 - 8.7) | 2.7 (1.1 - 6.4) | 1.2 (0.3 - 4.5) | -3.0 |
| Cyprus | 5.7 (2.1 - 14.4) | 17.1 (10.0 - 27.7) | 1.5 (0.2 - 9.8) | -4.2 |
| Czech Republic | 5.0 (2.5 - 9.7) | 3.9 (1.6 - 9.3) | 4.2 (1.6 - 10.8) | -0.8 |
| Denmark | 1.8 (0.4 - 6.9) | 2.3 (0.7 - 7.0) | 1.0 (0.1 - 6.4) | -0.8 |
| Estonia | 4.1 (1.5 - 10.5) | 2.7 (1.1 - 6.5) | 1.8 (0.6 - 5.5) | -2.3 |
| Finland | No observation | 0.4 (0.1 - 3.1) | No observation | Insufficient data |
| France | 1.3 (0.3 - 5.0) | 3.3 (1.3 - 7.9) | 1.3 (0.2 - 8.7) | 0.0 |
| Germany | 6.5 (3.5 - 11.8) | 14.8 (10.7 - 20.2) | 1.8 (0.7 - 4.5) | -4.7 |
| Greece | 1.8 (0.6 - 5.6) | 1.9 (0.6 - 6.0) | No observation | Insufficient data |
| Hungary | 1.7 (0.4 - 6.7) | 1.3 (0.2 - 8.8) | No observation | Insufficient data |
| Ireland | No observation | 4.6 (2.4 - 8.8) | 0.7 (0.1 - 4.7) | Insufficient data |
| Italy | No observation | 1.2 (0.3 - 4.8) | 4.2 (1.9 - 9.1) | Insufficient data |
| Latvia | 9.0 (4.8 - 16.3) | 4.1 (1.9 - 8.8) | 1.5 (0.4 - 5.7) | -7.5 |
| Lithuania | 3.0 (1.1 - 8.2) | 0.6 (0.1 - 4.4) | 0.6 (0.1 - 4.1) | -2.4 |
| Luxembourg | 6.3 (2.3 - 15.8) | 18.5 (10.6 - 30.3) | 2.5 (0.6 - 9.6) | -3.8 |
| Malta | No observation | 2.8 (0.4 - 17.1) | No observation | Insufficient data |
| Netherlands | 0.7 (0.1 - 4.8) | 1.8 (0.4 - 7.5) | 0.4 (0.1 - 3.1) | -0.3 |
| Poland | 0.8 (0.1 - 5.1) | 1.3 (0.3 - 5.8) | 2.1 (0.5 - 8.1) | +1.3 |
| Portugal | 0.5 (0.1 - 3.5) | 0.6 (0.1 - 4.1) | 0.5 (0.1 - 3.6) | 0.0 |
| Romania | 1.3 (0.4 - 4.2) | 1.7 (0.6 - 4.3) | 0.5 (0.1 - 3.4) | -0.8 |
| Slovakia | 1.5 (0.4 – 6.0) | 1.2 (0.3 - 4.5) | No observation | Insufficient data |
| Slovenia | 1.8 (0.4 – 7.0) | 4.2 (2.1 - 8.2) | 0.8 (0.1 - 5.5) | -1.0 |
| Spain | 1.1 (0.3 - 4.4) | 5.6 (3.0 - 10.0) | 0.5 (0.1 - 3.5) | -0.6 |
| Sweden | 1.9 (0.3 - 9.5) | 0.9 (0.2 - 3.7) | No observation | Insufficient data |
| **EU** | **2.5 (1.8 - 3.5)** | **5.1 (4.1 - 6.4)** | **1.8 (1.2 - 2.7)** | **-0.7** |

**Table S7: Prevalence of Current E-cigarette Use in Each EU MS across Waves**

| **Current Use –**  **E-cigarettes** | **Wave** | | | **Change (2017-2023)** |
| --- | --- | --- | --- | --- |
|  | **2017** | **2020** | **2023** |  |
| **Country** | **% (95% CI)** | **% (95% CI)** | **% (95% CI)** | **%** |
| Austria | 5.1 (2.4 - 10.8) | 6.2 (3.0 - 12.5) | 2.6 (1.0 - 6.6) | -2.5 |
| Belgium | 3.0 (1.2 - 6.9) | 3.7 (1.3 - 10.0) | 10.4 (7.4 - 14.5) | +7.4 |
| Bulgaria | 0.6 (0.1 - 4.0) | 1.1 (0.3 - 4.4) | 7.2 (4.1 - 12.4) | +6.6 |
| Cyprus | 4.0 (1.2 - 12.0) | 4.4 (1.4 - 13.2) | 6.8 (2.8 - 15.7) | +2.8 |
| Czech Republic | 1.3 (0.3 - 5.1) | 3.2 (1.4 - 6.8) | 8.2 (4.0 - 15.9) | +6.9 |
| Denmark | 1.4 (0.2 - 9.0) | 2.7 (0.8 - 8.3) | 3.6 (1.1 - 11.0) | +2.2 |
| Estonia | 5.4 (2.2 - 12.6) | 4.8 (2.1 - 10.5) | 18.0 (13.4 - 23.9) | +12.6 |
| Finland | 2.4 (0.8 - 7.1) | 1.1 (0.2 - 7.7) | No observation | Insufficient data |
| France | 0.9 (0.2 - 3.9) | 5.1 (2.5 - 10.3) | 9.5 (4.8 - 17.7) | +8.6 |
| Germany | 2.6 (1.1 – 6.0) | 2.5 (1.0 - 5.9) | 3.8 (1.9 - 7.6) | +1.2 |
| Greece | 4.9 (2.4 - 9.8) | 2.1 (0.8 - 5.4) | 3.6 (1.6 - 7.8) | -1.3 |
| Hungary | 1.0 (0.1 - 6.4) | No observation | 3.8 (1.4 - 9.8) | +2.8 |
| Ireland | 0.4 (0.1 - 2.4) | 7.1 (4.1 - 12.1) | 7.0 (3.8 - 12.4) | +6.6 |
| Italy | 0.5 (0.1 - 3.6) | 2.7 (1.1 - 6.5) | 2.1 (0.7 - 6.3) | +1.6 |
| Latvia | 2.5 (0.7 - 8.2) | 3.6 (1.5 - 8.4) | 16.3 (10.9 - 23.6) | +13.8 |
| Lithuania | 2.1 (0.5 - 8.2) | 6.4 (3.3 – 12.0) | 14.2 (9.0 - 21.8) | +12.1 |
| Luxembourg | 2.7 (0.7 - 10.1) | 0.8 (0.1 - 5.4) | 4.2 (0.9 - 16.6) | +1.5 |
| Malta | No observation | 2.0 (0.3 - 13.1) | 7.9 (2.5 - 22.7) | Insufficient data |
| Netherlands | 0.7 (0.1 - 4.6) | No observation | 4.4 (1.6 - 11.2) | +3.7 |
| Poland | 2.4 (0.9 - 6.5) | 4.2 (1.7 - 9.8) | 10.6 (5.5 - 19.5) | +8.2 |
| Portugal | 2.8 (1.2 - 6.6) | No observation | No observation | Insufficient data |
| Romania | No observation | 0.9 (0.2 - 3.5) | 0.5 (0.1 - 3.2) | Insufficient data |
| Slovakia | No observation | 1.3 (0.3 - 4.9) | 5.4 (2.5 - 11.4) | Insufficient data |
| Slovenia | 2.1 (0.5 - 7.9) | 0.6 (0.1 - 3.9) | 9.1 (5.1 - 15.9) | +7.0 |
| Spain | 0.4 (0.1 - 3.0) | 1.9 (0.6 - 5.9) | 3.2 (1.5 - 7.0) | +2.8 |
| Sweden | 0.6 (0.1 - 4.1) | No observation | 0.6 (0.1 - 4.0) | 0.0 |
| **EU** | **1.6 (1.1 - 2.3)** | **2.8 (2.1 - 3.9)** | **5.3 (4.0 - 6.8)** | **+3.7** |

**Table S8: Adjusted prevalence ratios (aPRs) of alternative definitions of ever and current use of waterpipe among individuals aged 15–30 years in the European Union (2012–23).**

|  | **Ever Regular Use** | | **Ever Use** (experimentation) | | **Current Use**  (including <monthly) | | **Current Daily Use** | |
| --- | --- | --- | --- | --- | --- | --- | --- | --- |
|  | **Weighted  prevalence (%)** | **aPRs (95% CI)** | **Weighted  prevalence (%)** | **aPRs (95% CI)** | **Weighted  prevalence (%)** | **aPRs (95% CI)** | **Weighted  prevalence (%)** | **aPRs (95% CI)** |
| **Waterpipe** | | | | | | | | |
| Year |  |  |  |  |  |  |  |  |
| 2012 | 11.4% | **0.82 (0.67-1.00)** | 22.4% | **1.17 (1.04-1.31)** | **/** | **/** | **/** | **/** |
| 2014 | 13.2% | 0.92 (0.80-1.07) | 25.2% | **1.21 (1.07-1.38)** | **/** | **/** | **/** | **/** |
| 2017 (ref.) | 12.9% | 1.00 | 18.5% | 1.00 | 6.5% | 1.00 | 0.3% | 1.00 |
| 2020 | 14.6% | 1.25 (0.95-1.65) | 20.2% | 1.13 (0.93-1.38) | 8.5% | 1.34 (0.97-1.86) | 0.6% | 2.01 (0.93-4.34) |
| 2023 | 5.5% | **0.45 (0.33-0.63)** | 10.0% | **0.50 (0.40-0.63)** | 4.0% | **0.54 (0.38-0.78)** | 0.2% | 0.56 (0.20-1.59) |
| **E-cigarettes** | | | | | | | | |
| Year |  |  |  |  |  |  |  |  |
| 2012 | 3.0% | **0.40 (0.30-0.54)** | 9.5% | **0.45 (0.38-0.54)** | **/** | **/** | **/** | **/** |
| 2014 | 5.6% | **0.70 (0.54-0.89)** | 14.2% | **0.74 (0.65-0.83)** | **/** | **/** | **/** | **/** |
| 2017 (ref.) | 6.2% | 1.00 | 18.6% | 1.00 | 2.1% | 1.00 | 1.0% | 1.00 |
| 2020 | 7.8% | **1.34 (1.10-1.63)** | 17.0% | 1.15 (1.00-1.32) | 2.3% | **1.49 (1.06-2.09)** | 1.5% | **1.99 (1.28-3.09)** |
| 2023 | 9.9% | **2.12 (1.56-2.87)** | 17.7% | 1.13 (0.94-1.35) | 5.3% | **3.84 (2.44-6.03)** | 3.4% | **6.45 (3.59-11.59)** |

Data are adjusted prevalence ratios (aPRs) with 95% CIs. Models were adjusted for all sociodemographic covariates (gender, age category, place of residence, difficulty in paying bills), smoking status, Actual Individual Consumption, and Tobacco Control Scale score. The 2017 survey wave serves as the reference category. Values in **bold** indicate statistical significance at p < 0.05. aPR = adjusted prevalence ratio; CI: Confidence interval. “Ever regular use” classifies respondents who have only tried the product once or twice as never users. “Ever use (experimentation)” was computed against “never use”; respondents reporting having tried waterpipes or e-cigarettes only once or twice were classified as “ever users” and other users were set to missing.

**Table S9: Adjusted prevalence ratios (aPRs) for ever-use of waterpipe and e-cigarettes among individuals aged 15–30 years in the European Union (2017–23), by sociodemographic group, after excluding 2020 data.**

|  | **2012** |  | **2014** |  | **2017** | **2023** | |  |
| --- | --- | --- | --- | --- | --- | --- | --- | --- |
|  | **Weighted  prevalence (%)** | **aPRs (95% CI)** | **Weighted  prevalence (%)** | **aPRs (95% CI)** | **Weighted  prevalence (%)** | **Weighted  prevalence (%)** | **aPRs (95% CI)** | |
|  |  |  |  |  | REF. Year |  |  | |
| **Ever Use of Waterpipe** | | | | | | | | |
| Overall | 31.2% | **1.10 (1.02-1.18)** | 35.0% | **1.15 (1.05-1.26)** | 29.1% | 14.9% | **0.50 (0.40-0.62)** | |
| Gender |  |  |  |  |  |  |  | |
| Woman | 25.7% | 1.10 (0.99-1.23) | 30.1% | **1.15 (1.02-1.29)** | 23.1% | 12.9% | **0.51 (0.40-0.64)** | |
| Man | 36.3% | 1.05 (0.97-1.15) | 39.6% | **1.11 (1.01-1.22)** | 34.7% | 16.9% | **0.55 (0.43-0.69)** | |
| Age (years) |  |  |  |  |  |  |  | |
| 15-17 | 25.2% | **1.36 (1.05-1.76)** | 24.2% | **1.30 (1.03-1.65)** | 19.0% | 4.0% | **0.48 (0.30-0.76)** | |
| 18-24 | 35.3% | 1.07 (0.97-1.17) | 38.9% | **1.15 (1.01-1.32)** | 31.7% | 16.8% | **0.55 (0.44-0.69)** | |
| 25-30 | 27.9% | 1.02 (0.91-1.14) | 33.6% | 1.04 (0.96-1.13) | 28.6% | 15.7% | **0.55 (0.44-0.69)** | |
| Area of Residence |  |  |  |  |  |  |  | |
| Rural | 25.2% | 0.92 (0.77-1.10) | 33.5% | 1.10 (0.93-1.30) | 27.2% | 9.7% | **0.53 (0.39-0.72)** | |
| Urban | 33.9% | **1.14 (1.06-1.23)** | 35.6% | **1.15 (1.06-1.25)** | 29.8% | 16.8% | **0.52 (0.43-0.64)** | |
| Difficulty Paying Bills |  |  |  |  |  |  |  | |
| Almost Never/Never | 33.2% | 1.06 (0.96-1.18) | 35.7% | 1.10 (0.97-1.26) | 30.8% | 12.8% | **0.46 (0.37-0.57)** | |
| Most of the Time/ From Time to Time | 28.3% | 1.11 (0.99-1.25) | 34.0% | **1.15 (1.02-1.30)** | 26.3% | 18.8% | **0.67 (0.51-0.87)** | |
| **Ever Use of E-Cigarettes** | | | | | | | | |
| Overall | 12.2% | **0.47 (0.41-0.54)** | 19.0% | **0.75 (0.68-0.84)** | 23.6% | 25.8% | **1.24 (1.07-1.43)** | |
| Gender |  |  |  |  |  |  |  | |
| Woman | 9.8% | **0.47 (0.39-0.56)** | 15.9% | **0.76 (0.66-0.56)** | 19.1% | 25.5% | **1.40 (1.17-1.68)** | |
| Man | 14.5% | **0.47 (0.40-0.55)** | 21.8% | **0.75 (0.66-0.85)** | 28.0% | 26.2% | 1.13 (0.97-1.31) | |
| Age (years) |  |  |  |  |  |  |  | |
| 15-17 | 8.6% | **0.43 (0.31-0.60)** | 12.7% | 0.75 (0.56-1.00) | 18.3% | 16.5% | 1.30 (0.94-1.81) | |
| 18-24 | 13.0% | **0.47 (0.40-0.56)** | 19.8% | **0.74 (0.64-0.86)** | 25.9% | 31.5% | **1.29 (1.13-1.48)** | |
| 25-30 | 12.4% | **0.48 (0.41-0.57)** | 19.9% | **0.77 (0.68-0.86)** | 22.5% | 21.4% | 1.17 (0.96-1.42) | |
| Area of Residence |  |  |  |  |  |  |  | |
| Rural | 11.4% | **0.40 (0.33-0.50)** | 18.6% | **0.69 (0.59-0.80)** | 24.5% | 24.0% | 1.16 (0.93-1.46) | |
| Urban | 12.6% | **0.50 (0.43-0.58)** | 19.1% | **0.78 (0.68-0.89)** | 23.3% | 26.5% | **1.28 (1.11-1.47)** | |
| Difficulty Paying Bills |  |  |  |  |  |  |  | |
| Almost Never/Never | 12.7% | **0.50 (0.42-0.58)** | 17.1% | **0.75 (0.65-0.86)** | 22.4% | 25.1% | **1.43 (1.25-1.64)** | |
| Most of the Time/ From Time to Time | 11.5% | **0.45 (0.37-0.54)** | 22.2% | **0.76 (0.66-0.54)** | 25.7% | 27.2% | 1.02 (0.85-1.23) | |

Data are adjusted prevalence ratios (aPRs) with 95% CIs. Models were stratified by each sociodemographic factor (gender, age category, place of residence, difficulty in paying bills), and adjusted for smoking status, Actual Individual Consumption, Tobacco Control Scale score, and all other listed covariates. The 2017 survey wave serves as the reference category. Values in **bold** indicate statistical significance at p < 0.05. aPR = adjusted prevalence ratio; CI: Confidence interval.

**Table S10: Adjusted prevalence ratios (aPRs) for current-use of waterpipe and e-cigarettes among individuals aged 15–30 years in the European Union (2017–23), by sociodemographic group, after excluding 2020 data.**

|  | **2017** | **2023** | |
| --- | --- | --- | --- |
|  | **Weighted  prevalence (%)** | **Weighted  prevalence (%)** | **aPRs (95% CI)** |
|  | REF. Year |  |  |
| **Current Use of Waterpipe** | | | |
| Overall | 2.5% | 1.8% | 0.77 (0.46-1.28) |
| Gender |  |  |  |
| Woman | 1.6% | 2.0% | 1.39 (0.75-2.59) |
| Man | 3.4% | 1.6% | **0.55 (0.34-0.88)** |
| Age (years) |  |  |  |
| 15-17 | 5.4% | 0.1% | 0.21 (0.02-2.52) |
| 18-24 | 2.5% | 2.2% | 0.71 (0.35-1.42) |
| 25-30 | 1.8% | 1.7% | 0.96 (0.30-1.15) |
| Area of Residence |  |  |  |
| Rural | 2.1% | 0.3% | **0.26 (0.12-0.55)** |
| Urban | 2.6% | 2.3% | 0.96 (0.54-1.70) |
| Difficulty Paying Bills |  |  |  |
| Almost Never/Never | 2.9% | 1.1% | 0.78 (0.36-1.51) |
| Most of the Time/ From Time to Time | 1.9% | 3.0% | 0.90 (0.41-1.95) |
| **Current Use of E-Cigarettes** | | | |
| Overall | 1.6% | 5.3% | **4.21 (2.73-6.49)** |
| Gender |  |  |  |
| Woman | 0.7% | 6.6% | **8.15 (4.57-14.62)** |
| Man | 2.4% | 4.0% | **2.77 (1.77-4.33)** |
| Age (years) |  |  |  |
| 15-17 | 1.2% | 4.4% | **4.19 (2.07-8.50)** |
| 18-24 | 1.4% | 6.2% | **4.69 (2.75-7.98)** |
| 25-30 | 1.9% | 4.3% | **3.89 (2.32-6.54)** |
| Area of Residence |  |  |  |
| Rural | 1.6% | 5.9% | **3.14 (1.75-5.62)** |
| Urban | 1.6% | 5.0% | **4.93 (3.02-8.06)** |
| Difficulty Paying Bills |  |  |  |
| Almost Never/Never | 1.4% | 4.7% | **4.31 (2.98-6.22)** |
| Most of the Time/ From Time to Time | 1.8% | 6.4% | **3.68 (1.73-7.84)** |

Data are adjusted prevalence ratios (aPRs) with 95% CIs. Models were stratified by each sociodemographic factor (gender, age category, place of residence, difficulty in paying bills), and adjusted for smoking status, Actual Individual Consumption, Tobacco Control Scale score, and all other listed covariates. The 2017 survey wave serves as the reference category. Values in **bold** indicate statistical significance at p < 0.05. aPR = adjusted prevalence ratio; CI: Confidence interval.
